# The effect of music-supported acceptance and commitment therapy on perceived stress and pain in cancer patients

**DOI:** 10.1590/1806-9282.20241027

**Published:** 2025-05-02

**Authors:** Mehtap Tan, Sibel Asi Karakaş, Mine Ekinci, Filiz Ersöğütçü, Asude Aksoy

**Affiliations:** 1 Atatürk University, Faculty of Nursing, Department of Internal Medicine Nursing – Erzurum, Turkey.; 2 Atatürk University, Faculty of Nursing, Department of Psychiatric Nursing – Erzurum, Turkey.; 3 Fırat University, Faculty of Health Sciences, Department of Nursing – Elazığ, Turkey.; 4 Fırat University, Faculty of Medicine, Department of Oncology – Elazığ, Turkey.

**Keywords:** Acceptance and commitment therapy, Cancer, Nursing, Pain, Psychological stress

## Abstract

**OBJECTIVE:**

The aim of this study was to investigate the effect of music-supported acceptance and commitment therapy on perceived stress and pain levels in cancer patients.

**METHODS:**

A total of 79 cancer patients participated in this controlled, pre-test/post-test quasi-experimental study (experimental group: n=29; control group: n=50). The intervention group received eight sessions of acceptance and commitment therapy with music, while the control group received standard care. Data were collected using the Perceived Stress Scale and the West Haven-Yale Multidimensional Pain Inventory.

**RESULTS:**

The post-test Perceived Stress Scale scores of the experimental group were statistically significantly lower compared to the control group (26.17±3.52 vs. 28.88±5.73, p<0.05), indicating a reduction in perceived stress. Additionally, there was a statistically significant difference in pain severity scores between the groups (9.62±2.33 in the experimental group vs. 8.06±3.14 in the control group, p<0.05). The effect size for stress reduction was moderate (Cohen's d=-0.54).

**CONCLUSION:**

This study revealed that a music-supported acceptance and commitment therapy reduced perceived stress, pain severity, and pain interference in cancer patients. Nurses should actively involve non-pharmacological methods in pain and stress management planning in collaboration with patients and their families. They should create a therapeutic environment and take necessary measures to enable patients to benefit from non-pharmacological interventions.

## INTRODUCTION

Cancers continue to be a significant concern in terms of morbidity and mortality among chronic diseases worldwide. According to the World Health Organization, cancer is one of the leading causes of death globally, ranking second after cardiovascular diseases in many countries, regardless of their economic status^1^. According to the Turkish Statistical Institute, cancer caused the death of 49,946 men and 27,022 women in Turkey in 2015^2^. Cancer-related pain, which affects over half of the cancer patients at any stage, remains one of the most significant issues, negatively impacting their quality of life^3^. Psychological interventions like mindfulness have been shown to reduce distress levels and enhance the quality of life in cancer patients. Interest in implementing acceptance and mindfulness-based approaches for adults has rapidly increased in recent years. Studies suggest that these approaches are effective in improving physical health, managing stress, and aiding recovery^4^. Acceptance and commitment therapy (ACT) is a contemporary therapeutic approach that emphasizes accepting and committing to changes in response to traumatic events. Recent studies have demonstrated the significant role of therapies like ACT in enhancing the quality of life for cancer patients. The core principles of ACT provide a robust framework for effective cancer management interventions^5^.

In the literature, there is a limited number of studies specifically examining the effectiveness of music-supported ACT in managing stress and pain among cancer patients. Recent research over the past few years has demonstrated the psychosocial benefits of ACT in managing chronic diseases, but there is a notable lack of data regarding its combination with music in oncology settings. Therefore, a more thorough investigation of the impact of music-supported ACT in this context is needed. This study aims to fill this gap and provide further insights into the effectiveness of non-pharmacological interventions for cancer patients.

## METHODS

### Study design

This is a controlled pre-test/post-test quasi-experimental study.

### Study population and sample

The study population consisted of all patients diagnosed with cancer, who received inpatient treatment between June 2019 and June 2020 and met the inclusion criteria for the study. All patients who met the study inclusion and exclusion criteria were randomly assigned to the intervention group (clinical treatment and practices+ACT and music) or the control group (clinical treatment and practices only) in a 1:2 ratio. The study was completed with 79 participants (29 in the experimental group and 50 in the control group).

Inclusion criteria:

- Agreeing to participate in the study;
- Willing to collaborate and communicate;
- Being 18 years old and above;
- Being diagnosed with cancer and undergoing treatment at the oncology clinic.

Exclusion criteria:

- Having psychotic symptoms;
- Having had a diagnosis of cognitive impairment;
- Having a lack of cognitive competence to continue the sessions during therapy.

### Intervention

#### Music concert

The researcher provided patients with a portable MP3 player and headphones as the music-listening device. Patients in the experimental group listened to relaxing music for 30 min, featuring wave sounds accompanied by harp and violin melodies from the third section of the "Relaxation Exercises CD" prepared by the Turkish Psychological Association. This music was specifically chosen for its calming effects.

#### Acceptance and commitment therapy

The therapy was administered by the researcher in a total of eight sessions, each lasting 45–50 min, following the protocol prepared according to the study objectives.

#### Data collection

The data were collected through face-to-face interviews conducted by the researchers using a Personal Information Form, the Perceived Stress Scale (PSS), and the West Haven-Yale Multidimensional Pain Inventory (WHYMPI). The interviews were conducted in two stages: a pre-test before the intervention and a post-test at a 2-month follow-up.

- Personal Information Form: This form was prepared by the researchers and consists of questions to determine the patients’ sociodemographic characteristics, as well as disease- and treatment-related features, including age, marital status, education level, type of cancer, and treatment modalities.
- PSS: The PSS was developed by Cohen et al. and consists of a total of 14 items designed to measure the extent to which certain situations in a given person's life are perceived as stressful. This is a 5-point Likert-type scale, scoring from "never (0)" to "very often (4)." Seven items contain positive statements and are scored in reverse. The total scale scores range from 0 to 56: Higher scores indicate greater perceived stress^6,7^. In this study, the Cronbach's alpha internal consistency coefficient for the PSS was found to be 0.64.
- WHYMPI: The WHYMPI was developed by Kerns et al. to assess relevant dimensions arising from the cognitive-behavioral theory for individuals with chronic pain. The scale is used to evaluate various clinical pains, such as cancer pain, fibromyalgia, headaches, temporomandibular disorders, chronic back pain, and chronic neck pain. It consists of three sections: "pain experience," "responses from significant others," and "daily activities," comprising a total of 60 items^8^. In this study, the Cronbach's alpha internal consistency coefficient for the WHYMPI was found to be 0.78.

### Data analysis

The data were analyzed using the IBM SPSS V21 software package. The Shapiro-Wilk test was used to assess whether the data had a normal distribution. The analysis results were presented using mean±standard deviation and median (minimum–maximum) for quantitative data and frequency for categorical data. A p-value of<0.05 was considered statistically significant. The chi-square test was used to compare descriptive characteristics between the groups, the independent t-test for comparing mean scale scores of the groups, least significant difference for advanced analysis, and repeated-measures tests for comparing mean scale scores within the groups. Before conducting the statistical analyses, the demographic and health-related characteristics of the experimental and control groups were compared. It was found that there were no statistically significant differences between the groups in terms of key variables, such as age, gender, education level, marital status, and treatment methods (p>0.05). These results indicate that the groups were homogeneous, minimizing the potential influence of confounding variables.

### Ethics

For conducting the study, ethical approval was obtained from the Ethics Committee of the Faculty of Nursing, Atatürk University (reference number: 2019-1/13, date: 29.01.2019), and institutional permission was obtained from Firat University Hospital (number: 19003918/604.02, date: 13.03.2019-317546) where the study was conducted. Informed verbal and written consent was obtained from all patients who agreed to participate in the study. This study was conducted in accordance with the Declaration of Helsinki and complied with the ethical standards.

## RESULTS

Table 1 compares the patients’ demographic characteristics and health characteristics. There was no statistically significant difference between the groups’ demographic characteristics and health characteristics (p>0.05); thus, the groups were homogeneous in terms of demographic characteristics and health characteristics.

**Table 1:** Comparison of the descriptive characteristics and health characteristics of the groups.

Characteristics		Experimental (n=29)		Control (n=50)		Test and p-value
		n	%	n	%	
Age						
	20–54 years	11	38.0	20	40.0	**χ** ^2^=3.84 p=0.14
	55–64 years	13	44.8	13	26.0	
	65 years and above	5	17.2	17	34.0	
Gender						
	Female	18	62.1	29	58.0	**χ** ^2^=0.12 p=0.72
	Male	11	37.9	21	42.0	
Education level						
	Illiterate	10	34.5	12	24.0	**χ** ^2^=1.06 p=0.78
	Only literate	8	27.6	16	32.0	
	Secondary education	5	17.2	11	22.0	
	High school and above	6	20.7	11	22.0	
Marital status						
	Married	20	69	42	84	**χ** ^2^=2.45 p=0.11
	Single	9	31	8	16	
Occupation						
	Homemaker	14	48.3	27	54.0	**χ** ^2^=1.00 p=0.60
	Retired	8	27.6	9	18.0	
	Other	7	24.1	14	28.0	
Number of children						
	2 or fewer	8	27.6	12	24.0	**χ** ^2^=1.69 p=0.42
	3–4	8	27.6	21	42.0	
	5 and more	13	44.8	17	34.0	
Health status						
	Good	3	10.3	7	14.0	**χ** ^2^=0.76 p=0.68
	Moderate	18	62.1	26	52.0	
	Poor	8	27.6	17	34.0	
Cancer in the family						
	Yes	12	41.4	36	72.0	**χ** ^2^=1.48 p=0.22
	No	17	58.6	14	28.0	
Presence of helper						
	Yes	26	89.7	44	88.0	**χ** ^2^=1.00 p=0.56
	No	3	10.3	6	12.0	
Presence of metastases						
	Yes	7	24.1	7	14.0	**χ** ^2^=1.29 p=0.25
	No	22	75.9	43	86.0	
Treatment methods						
	Chemotherapy	11	38.0	25	50.0	**χ** ^2^=1.87 p=0.39
	Radiotherapy	3	10.3	2	4.0	
	Multiple treatment modalities	15	51.7	23	46.0	

Table 2 compares the groups’ pre- and post-test mean scores of WHYMPI and PSS. The pre-test mean score of pain severity was 9.82±2.53 for patients in the experimental group and 8.06±3.14 for those in the control group; the difference between the groups’ mean scores was statistically significant (p<0.05). There was no statistically significant difference between the groups’ pre-test mean scores of other WHYMPI subscales, including pain interference, support of relatives, self-control, negative mood, punishing responses, solicitous responses, distracting responses, household chores, outdoor work, activities away from home, social activities, and general activities (p>0.05). In addition, the pain severity post-test mean score was 9.62±2.33 for patients in the experimental group and 8.06±3.14 for those in the control group, and the difference between the groups’ mean scores was statistically significant (p<0.05). There was no statistically significant difference between the groups’ other WHYMPI subscale post-test mean scores, including pain interference, support of relatives, self-control, negative mood, punishing responses, solicitous responses, distracting responses, household chores, outdoor work, activities away from home, social activities, and general activities (p>0.05). The pre-test mean score of PSS was 29.00±3.78 for patients in the experimental group and 28.06±6.27 for those in the control group, and the difference between the groups’ mean scores was not statistically significant (p>0.05). However, the PSS post-test mean score was 26.17±3.52 for patients in the experimental group and 28.88±5.73 for those in the control group, and the difference between the groups’ mean scores was statistically significant (p<0.05).

**Table 2:** Comparison of the intergroup WHYMPI and PSS mean scores.

	Groups	WHYMPI													PSS
		Pain experience					Responses from significant others			Daily activities					
		Pain interference	Support of relatives	Pain severity	Self-control	Negative mood	Punishing responses	Solicitous responses	Distracting responses	Household chores	Outhoor work	Activities away from home	Social activities	General activities	
Pre-test	Experimental	31.18±10.56	10.28±2.78	9.82±2.53	5.77±2.34	7.81±2.52	5.72±3.24	20.84±8.81	8.37±3.63	3.60±4.46	2.00±3.43	2.13±2.67	2.06±2.64	11.08±12.48	29.00±3.78
	Control	28.52±8.76	9.89±3.79	8.06±3.14	5.00±2.25	8.04±2.92	6.56±4.85	19.24±8.14	9.74±4.37	5.90±6.50	4.41±5.89	4.23±4.66	4.58±4.95	21.68±21.53	28.06±6.27
	Test and p-value	t=−1.20, p=0.23	Z=−0.15, p=0.98	t=0.37, **p=0.01**	Z=−1.77, p=0.07	Z=−0.43, p=0.66	Z=−0.44, p=0.65	t=−0.81, p=0.41	t=0.22, p=0.13	Z=−1.16, p=0.24	Z=−1.60, p=0.10	Z=−1.53, p=0.12	Z=−1.73, p=0.08	Z=−1.81, p=0.07	Z=−1.43, p=0.15
Post-test	Experimental	30.62±10.22	10.28±2.78	9.62±2.33	5.77±2.34	7.81±2.52	5.72±3.24	20.84±8.81	8.37±3.63	6.29±3.56	3.11±3.11	2.13±2.67	2.06±2.64	14.87±11.22	26.17±3.52
	Control	28.52±8.76	9.89±3.79	8.06±3.14	5.00±2.25	8.04±2.99	6.56±4.85	19.24±8.13	9.74±4.37	5.90±6.50	4.41±5.89	4.23±4.66	4.58±4.95	21.68±21.51	28.88±5.73
	Test and p-value	t=−0.96, p=0.33	Z=−0.15, p=0.98	t=0.16, **p=0.01**	Z=−1.77, p=0.07	Z=−0.40, p=0.68	Z=−0.45, p=0.64	t=−0.81, p=0.41	t=0.22, p=0.13	Z=−1.42, p=0.15	Z=−1.19, p=0.23	Z=−1.53, p=0.12	Z=−1.73, p=0.08	Z=−0.51, p=0.61	Z=−2.44, **p=0.01**

PSS: Perceived Stress Scale; WHYMPI: West Haven-Yale Multidimensional Pain Inventory; statistically significant values are denoted in bold.

Table 3 compares the pre- and post-test mean scores of intragroup WHYMPI and PSS for patients in the experimental group. The pre- and post-test mean scores of their pain interference were 31.18±10.56 and 30.62±10.22, respectively; the difference between their mean scores was statistically significant in favor of the post-test (p=0.001). The pre- and post-test mean scores of their pain severity were 9.82±2.53 and 9.62±2.33, respectively; the difference between their mean scores was statistically significant in favor of the post-test (p<0.005). The pre- and post-test mean scores of their household chores were 3.60±4.46 and 6.29±3.56, respectively; the difference between their mean scores was statistically significant in favor of the post-test (p<0.005). The pre- and post-test mean scores of their outdoor work were 2.00±3.43 and 3.11±3.11, respectively; the difference between their mean scores was statistically significant in favor of the post-test (p<0.005). The pre- and post-test mean scores of their general activities were 11.08±12.48 and 14.87±11.22, respectively; the difference between their mean scores was statistically significant in favor of the post-test (p<0.005).

**Table 3:** Intragroup comparison of the experimental group's West Haven-Yale Multidimensional Pain Inventory and Perceived Stress Scale mean scores.

Scales		Pre-test	Post-test	Test and p-value
		$\bar{X}$ ±SD	$\bar{X}$ ±SD	
WHYMPI	Pain interference	31.18±10.56	30.62±10.22	Z=−3.55, **p=0.001**
	Support of relatives	10.28±2.78	10.28±2.78	Z=0.001, p=1.00
	Pain severity	9.82±2.53	9.62±2.33	Z=−2.12, **p=0.03**
	Self-control	5.77±2.34	5.77±2.34	Z=0.001, p=1.00
	Negative mood	7.81±2.52	7.81±2.52	Z=0.001, p=1.00
	Punishing responses	5.72±3.24	5.72±3.24	Z=0.001, p=1.00
	Solicitous responses	20.84±8.81	20.84±8.81	Z=0.001, p=1.00
	Distracting responses	8.37±3.63	8.37±3.63	Z=0.001, p=1.00
	Household chores	3.60±4.46	6.29±3.56	Z=−4.58, **p=0.001**
	Outdoor work	2.00±3.43	3.11±3.11	Z=−4.23, **p=0.001**
	Activities away from home	2.13±2.67	2.13±2.67	Z=0.001, p=1.00
	Social activities	2.06±2.64	2.06±2.64	Z=0.001, p=1.00
	General activities	11.08±12.48	14.87±11.22	Z=−4.72, **p=0.001**
PSS		29.00±3.78	26.17±3.52	Z=−4.47, **p=0.001**

SD: standard deviation; PSS: Perceived Stress Scale; WHYMPI: West Haven-Yale Multidimensional Pain Inventory; statistically significant values are denoted in bold.

There was no statistically significant difference between the pre- and post-test mean scores (p>0.05) of their support of relatives, self-control, negative mood, punishing responses, solicitous responses, distracting responses, activities away from home, and social activities. The pre- and post-test PSS total mean scores of patients in the experimental group were 29.00±3.78 and 26.17±3.52, respectively; the difference between their mean scores was statistically significant in favor of the post-test (p<0.005).

## DISCUSSION

The results of this study, which aimed to examine the effects of music-supported ACT on perceived pain and stress levels in individuals diagnosed with cancer, were discussed in relation to the relevant literature.

In this study, the pre-test PSS total mean score was 29.00±3.78 for patients in the experimental group and 28.06±6.27 for those in the control group. A descriptive study on the relationship between perceived stress and hope in cancer patients with chemotherapy found their PSS mean score as 29.27±5.88^9^. The results of the present study are consistent with those in the literature. This study found a statistically significant difference between the groups’ pre- and post-test PSS mean scores, as well as between the experimental group's pre- and post-test intragroup PSS mean scores (p<0.05). The music-supported ACT intervention was effective in reducing the perceived stress levels of cancer patients. Music itself is not a stand-alone treatment, but when used for patients who are suffering, experiencing pain and stress, seeking help, and looking for a way to express themselves through music, it exhibits therapeutic properties^10^. The intervention of enjoyable music for cancer patients reduced their stress levels^11^. Stress begins as soon as patients learn about their cancer diagnosis, and they need psychological interventions. A study of 107 cancer patients with ACT reported a statistically significant difference between their pre- and post-test scores regarding the acceptance of the disease, stress management, well-being, and meaning in life^12^. A study about the effects of an eight-session ACT intervention on stress, anxiety, and depression in breast cancer patients found that the intervention led to a decrease in all areas^13^. A randomized controlled study of women with breast cancer reported that ACT improved their perceived stress, depression symptoms, and marital satisfaction^14^. A study on the effectiveness of ACT on quality of life and perceived stress in cancer patients has shown that the therapy significantly improved the quality of life and reduced perceived stress in cancer patients^15^.

The present study found a statistically significant difference in the experimental group's pre- and post-test mean scores of their pain severity and pain interference in favor of the post-test (p=0.001). The music-supported ACT intervention decreased both pain severity and interference among patients in the experimental group. The ACT is a psychosocial intervention with the potential to reduce symptom-related pain in cancer patients^16^. A study of women with breast cancer reported that ACT has positive effects on pain and anxiety^17^. A study of 70 patients with chemotherapy found that music interventions effectively reduced the severity of chemotherapy symptoms such as pain, fatigue, and nausea, and improved the comfort of patients undergoing chemotherapy^18^.

The present study also found a statistically significant difference in the experimental group's pre- and post-test mean scores of their household chores, general activities, and outdoor work in favor of the post-test (p<0.005). The music-supported ACT intervention increased daily activities among patients in the experimental group (Table 3). The ACT aims to increase awareness, reduce pain caused by diseases, alleviate exhaustion, address sleep problems and sedentary lifestyle, and evaluate post-traumatic growth^19^. The present study's results may reflect the effects of ACT.

## CONCLUSION AND RECOMMENDATIONS

While this study provides valuable insights into the effectiveness of music-supported ACT in reducing stress and pain among cancer patients, it has certain limitations. The small sample size and quasi-experimental design limit the generalizability of the findings. Future studies should aim to conduct randomized controlled trials with larger sample sizes to validate these results and explore the long-term effects of music-supported ACT, as well as its applicability to other patient populations or chronic conditions. Despite these limitations, the intervention was effective in decreasing perceived stress, pain severity, and pain interference in cancer patients. Therefore, when planning pain and stress management strategies, nurses should actively incorporate non-pharmacological methods into the care process, collaborating with both cancer patients and their families. Creating a therapeutic environment is essential to maximize the benefits of such interventions. Additionally, nurses should educate patients and their families on pain and stress management techniques, participate in developing multidisciplinary treatment plans, and take on evolving roles in patient care and education before, during, and after treatment. Supporting the use of evidence-based, non-pharmacological interventions and contributing to the establishment of standards for their implementation are also critical responsibilities for nursing professionals.
